# Geographic Variation in the Acoustic Traits of Greater Horseshoe Bats: Testing the Importance of Drift and Ecological Selection in Evolutionary Processes

**DOI:** 10.1371/journal.pone.0070368

**Published:** 2013-08-08

**Authors:** Keping Sun, Li Luo, Rebecca T. Kimball, Xuewen Wei, Longru Jin, Tinglei Jiang, Guohong Li, Jiang Feng

**Affiliations:** 1 Jilin Key Laboratory of Animal Resource Conservation and Utilization, Northeast Normal University, Changchun, China; 2 Key Laboratory for Wetland Ecology and Vegetation Restoration of National Environmental Protection, Northeast Normal University, Changchun, China; 3 Department of Biology, University of Florida, Gainesville, Florida, United States of America; 4 School of Life Science, Guizhou Normal University, Guiyang, China; University of Arkansas, United States of America

## Abstract

Patterns of intraspecific geographic variation of signaling systems provide insight into the microevolutionary processes driving phenotypic divergence. The acoustic calls of bats are sensitive to diverse evolutionary forces, but processes that shape call variation are largely unexplored. In China, *Rhinolophus ferrumequinum* displays a diverse call frequency and inhabits a heterogeneous landscape, presenting an excellent opportunity for this kind of research. We quantified geographic variation in resting frequency (RF) of echolocation calls, estimated genetic structure and phylogeny of *R. ferrumequinum* populations, and combined this with climatic factors to test three hypotheses to explain acoustic variation: genetic drift, cultural drift, and local adaptation. Our results demonstrated significant regional divergence in frequency and phylogeny among the bat populations in China's northeast (NE), central-east (CE) and southwest (SW) regions. The CE region had higher frequencies than the NE and SW regions. Drivers of RF divergence were estimated in the entire range and just the CE/NE region (since these two regions form a clade). In both cases, RF divergence was not correlated with mtDNA or nDNA genetic distance, but was significantly correlated with geographic distance and mean annual temperature, indicating cultural drift and ecological selection pressures are likely important in shaping RF divergence among different regions in China.

## Introduction

Understanding the origins and maintenance of phenotypic diversity is a fundamental goal of evolutionary biology [1]. The pattern of geographic variation in phenotypic characters can provide insights into the historical roles of drift and selection [2], [3], [4] including potential microevolutionary processes that drive population diversification and speciation [5], [6]. Acoustic signals in communication systems are one of the few diverse phenotypes that present opportunities for this kind of research. Phenotypic variation is readily quantifiable and easily accruable because of the combined effects of genotype and environmental factors [7], [8], [9].

Acoustic divergence can be influenced by a variety of factors such as geographic distance and landscape barriers [10], [11], divergent ecological selection [5], [12], [13], sexual selection [14], [15], genetic drift [5], [16], [17] and cultural drift [2], [6], [18], [19]. However, acoustic signal evolution is rarely explained by a single evolutionary force [16]. Instead, these factors may operate simultaneously in the same populations causing intraspecific geographic variation in call structure, or in separate populations in different parts of a species' range.

Patterns of geographic variation in acoustic signals occur in insects, frogs, birds and mammals (e.g., [6], [10], [20], [21], [22], [23]). Some researchers have shown that genetic drift plays an important role in acoustic divergence. Strong positive relationships between acoustic variation with geographic and genetic distances have been detected, suggesting the effect of stochastic processes (e.g., [16], [24]). If acoustic variation is only correlated with geographic (but not genetic) distances, this suggests that cultural drift has driven acoustic divergence for those taxa with a learned component to vocalizations (e.g., [2]). Alternatively, studies have also found that selection and adaptation played a major role in acoustic variation when acoustic variation is correlated with ecological factors (e.g., [12], [13]). When species face different selective pressures from food resources and environmental pressures, populations should exhibit different signal characteristics to adapt to different environmental situations. This can affect immigration between different environments and promote population divergence and ecological speciation [15].

Population genetic structure and dispersal routes could all play significant roles in leading to acoustic geographic variation. The effects of geographic isolation and population expansion could lead to species being distributed in different regions that vary in habitat and ecological characteristics, providing the possibility of either acoustic change due to drift in different populations, adaptation to different habitats, or both. Also, the population genetic structure may be related to call variation. Chen et al. [2] proposed that clarification of the relationship between patterns of echolocation call design and genetic structure required further work when they investigated the geographic variation of the *Rhinolophus monoceros* echolocation calls. However, investigations combining species' phylogenetic history with acoustic divergence are rare, though such studies are important to help understand the varying factors that can affect acoustic geographic variation.

Bats mainly use echolocation for orientation and locating food [25], [26], and frequency characteristics of calls may evolve in part by vocal learning [27]. Recent studies suggest that changes in the echolocation frequency of *Rhinolophus* bats are associated with assortative mating, reproductive isolation and resource use [28]. Thus, bats are an ideal model system to study the origin and maintenance of the geographic divergence of calls, which includes the role of acoustics in the speciation process. Currently, few studies have reported patterns of intraspecific geographic variation in echolocation calls. Although there have been investigations of echolocation calls in *R. monoceros* [2], *R. cornutus* [6], *R. pusillus* [13], *R. clivosus* [29], *Hipposideros ruber* [12], *H. larvatus* [22], *Craseonycteris thonglongyai* [11], and *Rhinonicteris aurantia* [30], [31], not all of these have incorporated genetic data. Thus the evolutionary forces and the meaning of intraspecific acoustic divergence are largely unexplored compared with the rich biodiversity of bats.

The greater horseshoe bat, *Rhinolophus ferrumequinum* (Rhinolophidae, *Rhinolophus*), emits constant frequency echolocation calls and is a widely distributed species covering most of the Palaearctic from Europe to China and Japan [32]. In China, *R. ferrumequinum* has a broad distribution from Jilin Province in northeast China to Yunnan Province in southwest China [33]. Genetic studies by Flanders et al. [34], [35] revealed divergent lineages of *R. ferrumequinum* in China, corresponding to different biogeographic regions. Bats were divided into eastern and southwestern groups, isolated by the Qinling Mountains, resulting in a restricted gene flow [34]. In these studies, one individual bat from northeastern China clustered with those from Japan, suggesting that *R. ferrumequinum* from northeast China could be a distinct lineage differing from the eastern and southwestern lineages. The bats inhabit a heterogeneous landscape with their distribution covering ca. 18° latitude. Previous studies reported that the dominant frequency of *R. ferrumequinum* was 69 kHz in Jilin Province, northeast China [36], and 75 kHz in Beijing City, east China [37], suggesting that Chinese *R. ferrumequinum* may exhibit a pattern of geographic variation in their echolocation calls. However, whether genetic drift, cultural drift, or adaptation to their environment (such as climate variation) drives the acoustic divergence of *R. ferrumequinum* remains unknown.

Our study quantified the resting frequency (RF) of echolocation calls emitted by *R. ferrumequinum* across China. We also investigated the climatic data of each locality and patterns of neutral genetic variation at the mitochondrial control region and microsatellite loci among populations to test evolutionary forces related to RF divergence. Our specific objectives were: (1) to determine whether there was detectable geographic variation in RF of *R. ferrumequinum*; and (2) to test the fit between observed patterns of RF geographic variation in *R. ferrumequinum* echolocation calls and those predicted by the following three hypotheses to explain RF divergence: (i) random genetic drift, (ii) cultural drift and (iii) acoustic adaption to different environments. To support the genetic drift hypothesis, we predicted that acoustic distance in RF would be correlated with genetic divergence at neutral loci (e.g., [5]). For the cultural drift hypothesis, we predicted that acoustic distance in RF would be related to geographic distance, but not related to genetic distance (e.g., [2], [13]). Finally, for the acoustic adaptation hypothesis, we predicted that RF divergence among populations would be related to climate factors because climatic factors (such as temperature and humidity) are known to have a direct influence on sound transmission (e.g., [5]). Our results will contribute to a better understanding of the importance of drift and selection in acoustic evolution.

## Materials and Methods

### Ethics Statement

According to the regulations of Wildlife Conservation of the People's Republic of China (Chairman Decree [2004] No. 24), permits are required only for those species included in the list of state-protected and region-protected wildlife species when it is collected. No specific permission was required because *R. ferrumequinum* is not an endangered or protected species, and is not on the list of state-protected or region-protected animal species. All sampling was conducted outside protected areas, and with permission of the forestry departments in the different areas. All field studies were approved by National Animal Research Authority in Northeast Normal University, China (approval number: NENU-20080416) and the Forestry Bureau of Jilin Province of China (approval number: [2006]178). Bats were captured using mist nets at cave exits. We then measured the bats, recorded the echolocation calls, sampled the wing membranes, and released them.

### Sample collection

**Figure 1:**
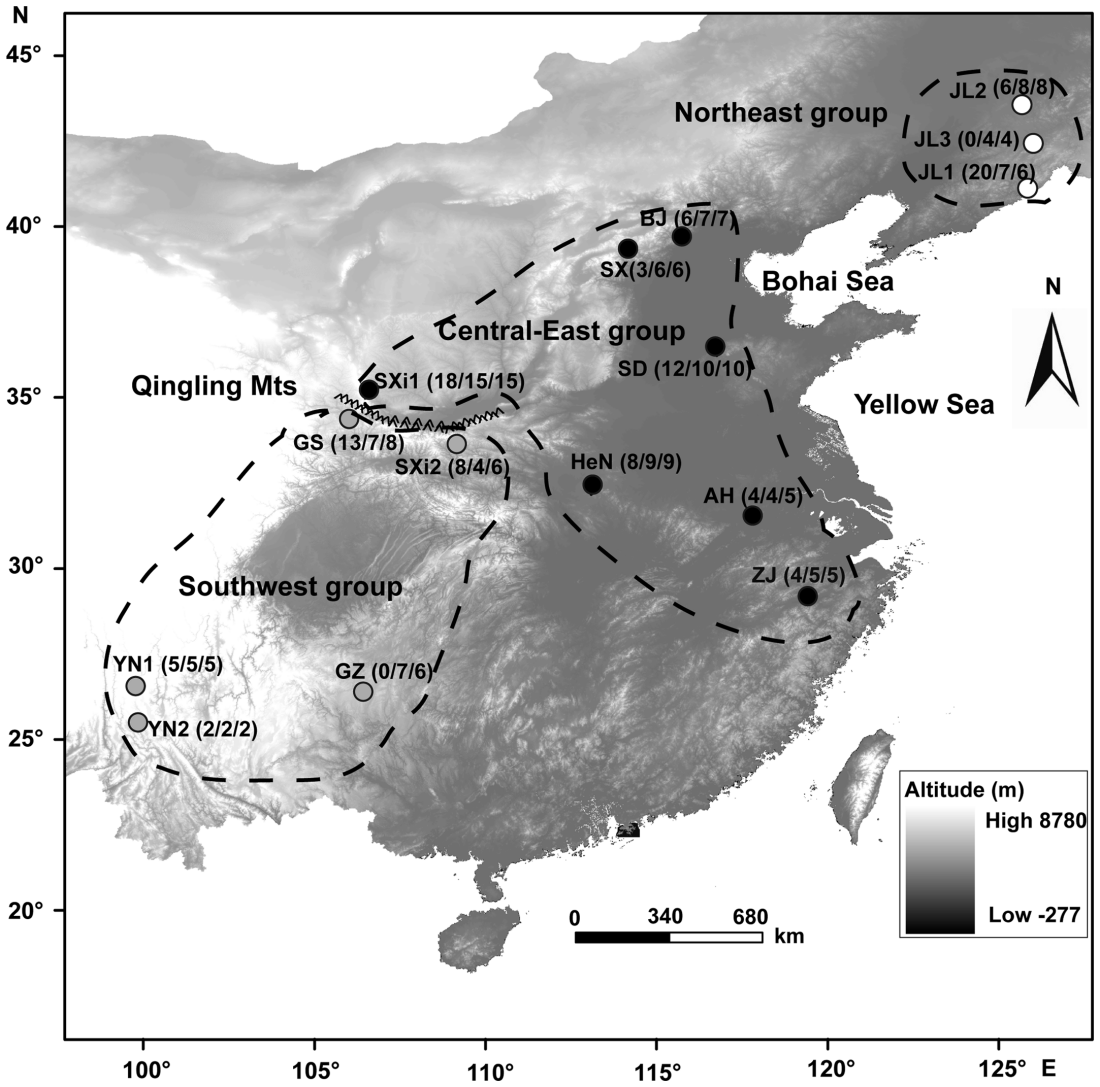
Sample localities of 15 *Rhinolophus ferrumequinum* populations used in this study. Populations were grouped according to the acoustic divergences and molecular phylogeny of *R. ferrumequinum*. Localities corresponding to the codes are given in Table 1. Sample sizes subjected to echolocation call/mtDNA/microsatellite DNA examination are in parentheses.

In China, 129 greater horseshoe bat samples were collected during 2006–2012 from 15 different localities (Fig. 1). Sampling strategies were different for echolocation calls, mtDNA and microsatellite DNA. For echolocation calls, 109 independent samples were recorded. For mtDNA, 100 samples were sequenced and 102 were examined using microsatellite DNA analysis. For each sampling locality, we determined the latitude and longitude using GPS (eTrex Vista) and then calculated the geographic distance matrixes for each locality.

MAPW- mean annual precipitable water, MARH- mean annual relative humidity, MAT- mean annual temperature, mRF- mean resting frequency, N- sample size.

### Echolocation call recording and analysis

Echolocation calls were recorded with a D980 Pettersson bat detector (Pettersson Electronik AB, Uppsala, Sweden; frequency response 8 and 160 kHz ±3.5 dB). This detector was positioned approximately 30 cm in front of hand-held bats. The recordings were transferred to a computer with a time expansion of ×10 using BatSound Pro 3.10 (sampling frequency of 441 kHz, 16 bit precision). The RF that the bats emitted under these conditions is the stable CF portion of the echolocation pulses emitted by the motionless bat, which coincides with the response frequency of the cochlear ‘acoustic fovea’ [38]. We selected a portion of a recording in which RF calls were continuously uttered. Using criteria as proposed by Russo et al. [39] and Jiang et al. [13], all high-quality calls within the selected portion of the recording were used for an individual. These criteria consisted of: (1) exclusion of the initial calls within a call series because these calls may show transient, lower frequency values before reaching the final RF level [40]; and (2) using the second call per group when calls were emitted in groups (doublets, triplets, etc.) [41]. From each individual, the 15 high-quality calls were measured for the CF component in the dominant second harmonic from power spectra of a call, and the mean value was used in the analysis. Within-individual variation was very small, the SD averaging 0.13 kHz, ranging from 0 to 0.30 kHz. Similar observations have been made in related species (e.g., [6], [30]).

To test for an effect of population and sex on RF, we tested for differences in RF among localities and between sexes using a general linear model (GLM) with type III sums of squares, and both variables were treated as fixed effects in the model. We performed a Mann-Whitney U test on the mean call frequency values obtained at each location. This allowed us to evaluate gender differences in RF at each locality when the individuals in each population were greater than three.

We used multidimensional scaling (MDS) to arrange acoustic distance between population pairs in a two-dimensional space. Variation in the RF of echolocation calls for males and females between different populations was calculated using Euclidean distances (SPSS Inc., version 16.0). MDS can be applied to any kind of distance matrix, and involves moving objects around in a defined space, using a number of dimensions, to maximizing the goodness-of-fit to the observed distance matrix. From the MDS plot, the RF of all populations clustered into three distinct groups. Therefore, *post-hoc* multiple comparison Tukey tests were used to test the differences among groups. To test the effect of group and sex on RF we used a GLM with group and sex as fixed effects in the model.

### Mitochondrial DNA sequencing and analysis

Samples for the genetic analysis were collected from bat wing membrane. Bat wing membrane (plagiopatagium) samples were acquired using biopsy punches (3 mm diameter) as outlined by Worthington Wilmer and Barratt [42]. The 3-mm holes in each wing heal in approximately 4 weeks [42]. Genomic DNA was extracted from the wing biopsies using the Animal Genomic DNA Isolation Kit (Sangon, China). For DNA amplification of control region, the primers P and E, and the polymerase chain reaction (PCR), we used methods as described in Wilkinson and Chapman [43]. Amplified products were purified and sequenced by Shanghai Sangon Biotechnology Co., Ltd. All *R. ferrumequinum* haplotypes and sequences were deposited in GenBank with accession numbers JN230556-JN230592.

All sequences were aligned using Clustal_X [44] and revised manually. Haplotype designation was conducted with Arlequin 3.0 [45]. Pairwise *F*_ST_ values between populations were calculated based on sequence information using the model specified by Tamura and Nei [46]. For the phylogenetic analyses, redundant haplotypes were removed from the data set.

Phylogenetic relationships were reconstructed using the maximum likelihood (ML) method implemented in PHYML v2.4.4 [47], neighbor-joining (NJ) and maximum parsimony (MP) in PAUP* 4.0 [48]. For PHYML, the starting tree was obtained with BIONJ [49]. The DNA substitution model that best fit the data was estimated using the Akaike information criterion using MODELTEST v3.06 [50]. HKY + Γ + I model of evolution was selected to estimate ML and NJ trees, accommodating rate variation among sites and including a proportion of invariable sites. The parameters were estimated and optimized during the search (transition/transversion  = 9.925, α-shape  = 0.653, I = 0.529). The MP tree was estimated using a heuristic search with 100 random additions, and complete tree-bisection-reconnection branch swapping for each iteration with equally weighted characters. Reliability of nodes was assessed using 1,000 bootstrap replications for all three methods [51]. Nodes that received ≥ 70% bootstrap support were considered well supported. Trees were rooted with sequences of *R. pearsoni* and *R. pusillus* (GenBank: EU053159 and DQ916121). A minimum-spanning network (MSN; [52]) for the observed haplotypes was generated using Arlequin. Analysis of molecular variance (AMOVA) was calculated in Arlequin with 1,000 permutations. Population structure was defined on the basis of phylogenetic clusters obtained from our analyses.

### Microsatellite data collection and analysis

Seven microsatellite loci, Reffer 15, 17, 19, 22, 24, 27 and 28, were amplified using fluorescently labeled primers. Primer sequences and PCR conditions followed those of Dawson et al. [53]. Amplified PCR products were analyzed using the ABI 3730 automated DNA sequencer, and Genemarker 1.5 was used to obtain allele designations. Analyses were performed by the Beijing Jinuoboshi Biotechnology Co., Ltd.

All loci were screened for null alleles and large allele dropouts using Micro-Checker v2.2.3 [54]. Tests for deviation from Hardy-Weinberg equilibrium were performed for each population, including a globally unified population using Arlequin. Genetic divergence was investigated using pairwise *F_ST_* values [55].

We analyzed the genetic structure of our data set using two different methods. First, we used a phylogenetic tree based on the shared allele distance between individuals and reconstructed using a NJ algorithm using Populations v1.2.30 [56]. Second, we used a Bayesian approach using Structure v2.3.1 [57]. The admixture model was used and the number of clusters (*K*) was based on the number of lineages recovered in the mtDNA analysis. For *K* = 3, 10 runs were performed with 1,000,000 iterations after 30,000 iterations were discarded as “burn-in”. The graphical display of the genetic structure was produced by DISTRUCT [58]. An AMOVA was performed in the same way as described for the mtDNA data analysis above.

### Climate data collection and analysis

We used climate data from each locality to summarize environmental features relevant to sound propagation. Temperature and humidity have a direct influence on sound transmission [5]. Therefore, we selected temperature and humidity as factors to describe environmental variation. To test for correlations between observed RF of echolocation calls and climate conditions at different localities, we obtained climate information for each locality covering the years 1980–2010 from the RNCEP, a package of functions in the open-source R language [59], including mean annual temperature (MAT), mean annual precipitable water (MAPW), and mean annual relatively humidity (MARH) (Table 1). Climatic distances of each climate factor between localities were summarized with dissimilarity matrices (Euclidean distances) using SPSS.

**Table 1:** Coordinates, climatic data, and resting frequency in echolocation calls of *Rhinolophus ferrumequinum* populations from China.

							Female		Male		All
Region	Population	Latitude (°N)	Longitude (°E)	MAP (kg/m^2^)	MAR (%)	MAT (°C)	N	mRF	N	mRF	mRF
Northeast	Ji'an, Jilin (JL1)	41.07	125.84	14.84	81.33	4.77	11	69.78±0.40	9	69.34±0.39	69.58±0.45
	Liuhe, Jilin (JL2)	42.38	126.00	13.68	79.22	3.80	3	68.68±0.38	3	68.54±0.13	68.61±0.26
	Shuangyang, Jilin (JL3)	43.52	125.67	-	-	-	-	-	-	-	-
Central-East	Lingqiu, Shanxi (SX)	39.33	114.30	12.90	59.97	7.24	3	75.92±0.30	0	-	75.92±0.30
	Beijing (BJ)	39.72	115.98	13.67	60.16	8.16	5	76.51±0.51	1	76.10±0.00	76.44±0.49
	Jinan, Shandong (SD)	36.45	116.95	19.98	60.17	12.99	7	77.14±0.36	5	76.20±0.49	76.75±0.63
	Baoji, Shaanxi (SXi1)	35.04	106.67	14.53	66.08	8.36	11	75.67±0.48	7	75.47±0.69	75.59±0.56
	Nanyang, Henan (HeN)	32.40	113.28	27.04	82.50	14.44	5	77.13±0.50	3	76.23±0.73	76.79±0.71
	Jinhua, Zhejiang (ZJ)	29.21	119.67	28.91	84.97	14.92	0	-	4	75.57±0.57	75.57±0.57
	Hanshan, Anhui (AH)	31.56	118.08	27.06	80.61	14.34	0	-	4	76.04±0.65	76.04±0.65
Southwest	Shangluo, Shaanxi (SXi2)	33.59	109.16	18.99	68.81	10.72	3	73.79±0.66	5	72.38±0.77	72.91±1.00
	Jianchuan, Yunnan (YN1)	26.52	99.77	15.09	81.27	8.62	0	-	5	72.77±0.56	72.77±0.56
	Tianshui, Gansu (GS)	34.33	106.01	16.70	70.61	7.84	5	73.95±0.34	8	73.03±0.79	73.38±0.79
	Guiyang, Guizhou (GZ)	26.35	106.42	-	-	-	-	-	-	-	-
	Xiangyun, Yunnan (YN2)	25.45	99.84	18.69	79.51	11.98	0	-	2	72.89±0.05	72.90±0.05

Values are given as mean ± SD. For JL3 and GZ populations, call data were not obtained.

### Correlation analysis

To assess whether differences in RF were related to geographic, genetic and climatic distance, we first calculated a dissimilarity matrix of acoustic distances using RF differences (kHz) between localities and calculated genetic distances using Slatkin's linearized *F_ST_* (given by *F_ST_*/(1– *F_ST_*)) [60]. We used pairwise Mantel tests as a priori choice of tests to assess important variables for the acoustic variation from three geographic and three climatic factors, respectively. For those important geographic and climatic factors, pairwise partial Mantel tests were used to test for associations between acoustic and geographic, genetic and climatic distances after removing the effects of genetic, climatic or geographic distance, respectively. All Mantel and partial Mantel tests used 10,000 permutations and were analyzed with PASSaGE v.2 [61]. We corrected critical a values for multiple Mantel and partial Mantel tests using Bonferroni adjustment [62].

## Results

### Echolocation call variation

**Figure 2:**
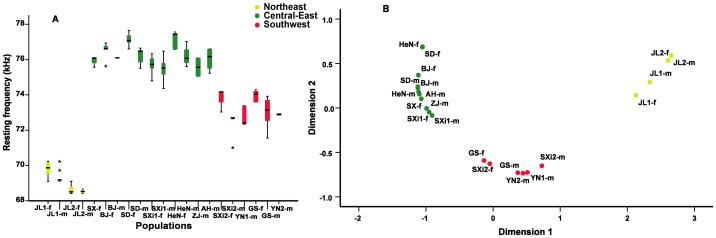
Variation in the resting frequency component of echolocation calls among *Rhinolophus ferrumequinum* populations in China. A. The resting frequency (RF) variation in echolocation calls. Populations with sex (female [f] and male [m]) are arranged on the X axis. For each box plot, the box represents the 0.25 quantile, median and 0.75 quantile. On either side of the box, the whiskers extend to the minimum and maximum values. The colors in the plot correspond to three groups in Figure 1. B. Multidimensional scaling plot of acoustic distance (measured as Euclidean distances by RF differences) between pairs of populations of adult males (m) and females (f).

We recorded and analyzed 1,635 calls from 109 adult *R. ferrumequinum* individuals comprising of 53 females and 56 males from 13 localities with 15 calls per bat. Across all samples, RF ranged from 68.41 to 77.65 kHz (*SD*  = 3.03) in females and from 68.41 to 77.05 kHz (*SD*  = 2.67) in males (Table 1, Fig. 2A). Our GLM model showed that both sex and population significantly influenced RF in *R. ferrumequinum*, however, the interaction between sex and population was not significant (Table 2). Female calls were significantly higher for each population when we compared frequencies within populations sampled using more than three individuals of each sex (Mann-Whitney U test: *P*<0.05 in all cases), except for JL2, SXi1 and HeN populations.

**Table 2:** Effect of Sex and Population on the resting frequency of *Rhinolophus ferrumequinum*.

GLM	*df*	MS	*F*	*P*
Factor				
Sex	1	7.116	25.149	<0.0001
Population	12	68.223	241.106	<0.0001
Sex × population	7	0.453	1.603	0.145
Error	88	0.283		

Tests used type III sum of squares. Sex and Population were considered as fixed effects. The model is significant and explains a large proportion of data variance (*F*_20_  = 153.063, *P*<0.0001, *r* ^2^  = 0.972).

We performed a two-dimensional MDS model to discriminate clusters of RF variation. The acoustic distances in RF for male and female bats within each population were estimated separately because of sexual dimorphism in RF. The first two dimensions extracted from the MDS model described nearly all the variation in RF among bat populations (*r* ^2^  = 0.9996). The MDS revealed three distinct geographic groups: the northeast (NE), the central-east (CE) and the southwest (SW) (Fig. 2B). Our results indicated that sex and group showed significant effects on RF. No interaction was detected between sex and group (Table 3), suggesting that the calls of both sexes responded in the same manner across groups. In this case each group included male and female individuals from the same population, which suggested that RF differences between sexes within populations were lower than those between groups. *Post-hoc* multiple comparison tests confirmed that the RF of *R. ferrumequinum* was significantly different between all groups (*P*<0.0001 in all cases). The CE showed the highest RF (76.16±0.77 kHz), while the NE group had the lowest RF (69.36±0.58 kHz). The RF in the SW group had an intermediate frequency (73.10±0.81 kHz). The NE group was restricted to northeast China, whereas the CE group was separated from the SW group by the Qinling Mountains (Fig. 1).

**Table 3:** Effect of Sex and Group on the resting frequency of *Rhinolophus ferrumequinum*.

GLM	*df*	MS	*F*	*P*
Factor				
Sex	1	10.408	23.129	<0.0001
Group	2	405.592	901.321	<0.0001
Sex × group	2	0.848	1.885	0.157
Error	103	0.450		

Tests used type III sum of squares. Sex and Group were considered as fixed effects. The model is significant and explains a large proportion of data variance (*F*_5_  = 375.454, *P*<0.0001, *r* ^2^  = 0.948).

### Population genetic analyses

**Figure 3:**
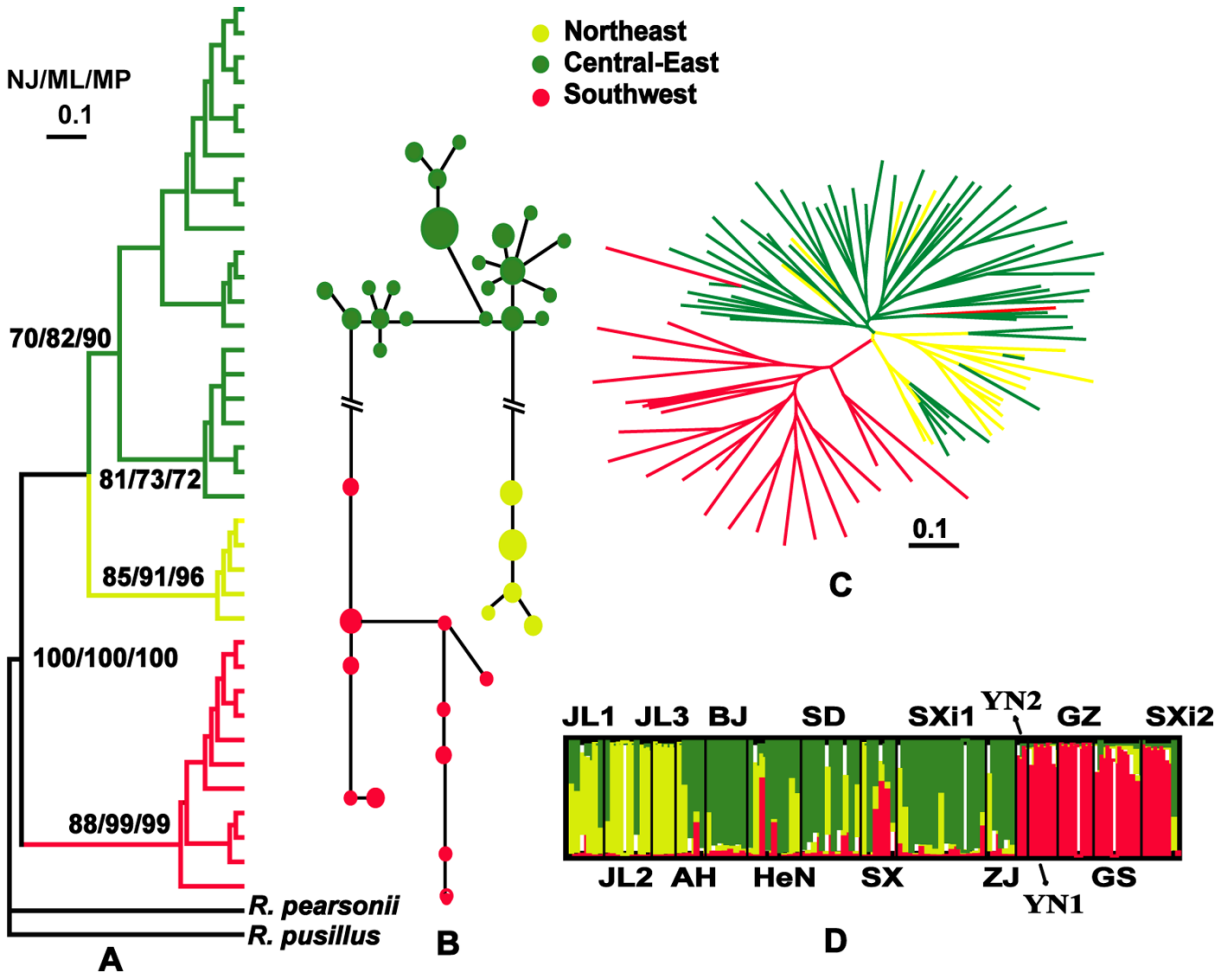
Phylogeny and haplotype among *Rhinolophus ferrumequinum* populations in China. A. Phylogenetic trees for 37 mtDNA haplotypes of *R. ferrumequinum*. Numbers above the tree branches are the bootstrap values for NJ, ML and MP methods. The node supports are given if bootstrap exceeded 70%. B. The minimum spanning network for the *R. ferrumequinum* haplotypes. The size of the shape is proportional to the frequency of that haplotype. C. Unrooted neighbor-joining tree reconstructed from shared allele distances based on microsatellite genotypes of all individuals in China. D. Bayesian cluster analyses with Structure (*K*  = 3) of 102 *R. ferrumequinum* samples based on seven microsatellites. Each vertical bar represents one individual and its probabilities of being assigned to clusters.

Amplification of approximately 477 bp of the mitochondrial DNA (mtDNA) control region recovered 37 haplotypes for 100 *R. ferrumequinum* from 15 populations. This included 13 populations with their frequency recorded and 2 populations (JL3 and GZ, Fig. 1) where frequency was not recorded (these were included to provide a better estimate of genetic structure). Phylogenetic reconstructions of the NJ, ML and MP analyses produced highly concordant trees. Three major monophyletic haplogroups in *R. ferrumequinum* were identified and designated as the NE, CE, and SW groups (Fig. 3A), which were congruent with those defined in RF (Fig. 2). The SW group at the basal position diverged first. The MSN parsimony network for the control region confirmed that three groups all differed and CE was from NE and SW by 27 and 31 mutational steps, respectively (Fig. 3B). The NE was closest (0.068±0.0058) to CE and more distant (0.087±0.0062) to SW.

A total of 102 individuals were genotyped and scored at seven nuclear DNA (nDNA) microsatellite loci. Across all sampling localities, the number of alleles per locus varied from 1 to 8 with mean number of alleles per locus at 2.3–4.4. All individual-based analyses of nDNA supported the presence of the three divergent evolutionary groups (NE, CE and SW) that were identified in the mtDNA data. However, 15 individuals (14.5%) between adjacent populations of different lineages were identified in unexpected groups based on their genotype (Fig. 3C). For example, two individuals from the SW region were assigned to CE, nine from CE to NE and four from NE to CE (Fig. 3C). Bayesian STRUCTURE analysis also showed three clusters at *K* = 3, corresponding to the three major groups (NE, CE and SW) identified in the mtDNA and nDNA trees (Fig. 3D). AMOVA was used to determine the proportion of genetic variance attributed to three major geographic areas (NE, CE and SW). In general, significant genetic heterogeneity was detected at various hierarchical levels (among groups, among populations within groups, and within populations) with both markers (Table 4). The mtDNA displayed higher differentiation at all three hierarchical levels than the microsatellite markers (Table 4).

**Table 4:** Analysis of molecular variance of *Rhinolophus ferrumequinum* in three geographic groups (NE, CE and SW).

Marker	Source of variation	*df*	Sum of squares	Variance component	Variation (%)	Fixation indices
mtDNA	Among groups	2	858.2	13.89	77.80	Φ_CT_: 0.778**
	Among populations within groups	12	233.8	2.82	15.81	Φ_SC_: 0.712**
	Within populations	85	97.1	1.14	6.40	Φ_ST_: 0.936**
Microsatellite	Among groups	2	59.1	0.43	16.32	Φ_CT_: 0.163**
	Among populations within groups	12	46.2	0.13	5.14	Φ_SC_: 0.215**
	Within populations	189	389.1	2.06	78.54	Φ_ST_: 0.163**

** *P*<0.001.

### Correlates of call divergence

For all *R. ferrumequinum* populations, Mantel tests showed that RF variation was significantly and positively associated with geographic distances (*r* = 0.36, *P* = 0.019, Table 5), even when controlling for mtDNA genetic distance (*r* = 0.37, *P* = 0.018, Table 5). RF also was significantly related with longitude, whether or not the effect of mtDNA, nDNA or MAT was removed (all cases, *P*≤0.018, Table 5). Likewise, a strong positive correlation occurred between RF and MAT in both the Mantel and partial Mantel tests (all cases, *P*≤0.013, Table 5). However, there was no significant correlation between RF and genetic distance. Significant pairwise positive correlations occurred between nDNA and all three geographic distance measures (all cases, *P*≤0.008, Table 5), even when RF distances were controlled (all cases, *P*≤0.014, Table 5). In contrast, there were no significant relationships found between mtDNA and geographic factors.

**Table 5:** Association between geographic distance in km, genetic divergence (*F_ST_*/1-*F_ST_*), climatic Euclidean distance and resting frequency.

		Total range		Within CE/NE regions	
Mantel tests		*r*	*P*	*r*	*P*
RF	geographic	0.36	**0.019**	0.59	**0.026**
	latitudinal	0.23	0.06	0.29	0.07
	longitudinal	0.41	**0.01**	0.62	**0.015**
	MAPW	−0.03	0.88	−0.10	0.34
	MARH	−0.09	0.54	−0.08	0.57
	MAT	0.44	**0.005**	0.49	**0.007**
	mtDNA	−0.03	0.86	0.19	0.57
	nDNA	0.23	0.09	0.49	0.03
mtDNA genetic	geographic	0.16	0.38	0.57	**0.002**
	latitudinal	0.07	0.70	0.27	0.13
	longitudinal	0.18	0.30	0.54	**0.025**
nDNA genetic	geographic	0.69	**0.0001**	0.45	**0.02**
	latitudinal	0.43	**0.008**	0.46	**0.004**
	longitudinal	0.69	**0.0001**	0.21	0.37
Partial Mantel test					
RF	geographic (mtDNA genetic)	0.37	**0.018**	0.60	**0.026**
	geographic (nDNA genetic)	0.29	0.04	0.47	0.07
	geographic (MAT)	0.28	0.04	0.42	0.07
	longitudinal (mtDNA genetic)	0.43	**0.009**	0.62	**0.019**
	longitudinal (nDNA genetic)	0.36	**0.018**	0.60	**0.017**
	longitudinal (MAT)	0.39	**0.018**	0.61	**0.015**
	MAT (geographic)	0.37	**0.013**	0.20	0.27
	MAT (latitudinal)	0.39	**0.008**	0.43	**0.024**
	MAT (longitudinal)	0.41	**0.007**	0.48	**0.008**
	mtDNA genetic (geographic)	−0.10	0.62	−0.22	0.48
	mtDNA genetic (longitudinal)	−0.12	0.58	−0.21	0.50
	nDNA genetic (geographic)	−0.02	0.87	0.31	0.25
	nDNA genetic (longitudinal)	−0.08	0.63	0.47	0.07
mtDNA genetic	geographic (RF)	0.19	0.32	0.58	**0.002**
	longitudinal (RF)	0.21	0.23	0.54	**0.014**
nDNA genetic	geographic (RF)	0.66	**0.0002**	0.23	0.31
	latitudinal (RF)	0.39	**0.014**	0.38	**0.024**
	longitudinal (RF)	0.67	**0.0001**	−0.13	0.79

Euclidean distance of echolocation calls in kHz between populations of *Rhinolophus ferrumequinum* in China. Geographic, longitudinal and MAT variables were chosen *a priori* as important factors and so were used to conduct the pairwise partial Mantel test. Results are shown as correlation coefficients of Mantel and partial Mantel tests. Controlled variables in partial Mantel tests are in parentheses. After Bonferroni correction [62], the *P* values that are significant in Mantel and partial Mantel tests, respectively, are shown in bold.

RF- resting frequency, MAPW- mean annual precipitable water, MARH- mean annual relative humidity, MAT- mean annual temperature.

Within the NE/CE region, the correlations between RF variation and geographic distance were similar to those within the total range. RF variation was significantly and positively associated with geographic distances (*r* = 0.59, *P* = 0.026, Table 5), even when mtDNA genetic distance was controlled for (*r* = 0.60, *P* = 0.026, Table 5). RF also was significantly related with longitude, whether or not the effect of mtDNA, nDNA or MAT was removed (all cases, *P*≤0.019, Table 5). We found a highly significant positive relationship between RF and MAT, even when the latitudinal or longitudinal distance was controlled (all cases, *P*≤0.024, Table 5). But RF and genetic (mtDNA or nDNA) distance showed no significant relationship. Additionally, mtDNA distance was significantly and positively associated with geographic and longitudinal distances, while nDNA distance was significantly associated with latitudinal distances (all cases, *P*≤0.025, Table 5).

## Discussion

In this study we sought to determine whether there was detectable geographic variation in RF of *R. ferrumequinum* in China, and if so, what factors might have played a major role in shaping that geographic variation in RF. We found substantial geographic variation in the echolocation call frequency of *R. ferrumequinum*. Our data support both cultural drift and adaptation to different climatic conditions as factors that contribute to the observed geographic variation in acoustic calls. We did not test two additional hypotheses that could contribute to geographic variation in calls – resource partitioning and interspecific interference. Resource partitioning of prey is unlikely as the range of wavelengths (4.46 to 4.90 mm; assuming a typical speed of sound of 340 m/s, an ambient temperature of 22 °C, and 80% humidity) is smaller that would be expected to forage for different prey sizes [41]. While interspecific competition in call frequencies has been observed between different bat species [11], [39], our study indicated no other sympatric bat calling at similar frequencies in any of the sampled populations (e.g., *Hipposideros armiger* has a lower calling frequency of ∼67–69 kHz).

### Geographic variation in echolocation frequency

The fine-scale analysis of RF variation conducted in this study revealed significant regional divergence in echolocation calls in *R. ferrumequinum* populations, indicating more variation than previously documented [36], [37]. The MDS plot showed that RF variation of all *R. ferrumequinum* populations studied clustered into three groups, NE, CE and SW, corresponding to biogeographic regions. The large differences in RF among groups suggested a significant acoustic variation in *R. ferrumequinum*.

The differences between males and females in RF should not have influenced the groups identified from the MDS since the variation between the sexes was relatively small and is less than that among groups (Fig. 3). The differences between males and females varied among populations. Males uttered significantly higher RFs than females in most (but not all) populations, in contrast to what is typical of rhinolophid bats in which females normally emit calls at a higher frequency than males (e.g., *R. hipposideros* [63], *R. cornutus* [6], [64], *R. monoceros* [2] and *R. capensis* [65]). However, the higher call frequency in females is not universal among rhinolophid bats [13], [66], consistent with our data. The marked overlap in RF between males and females in *R. ferrumequinum* suggests that call frequency is probably a poor signal for sex recognition [66].

The three groups identified from the RF of echolocation calls corresponded to the three phylogenetic groups, NE, CE and SW, identified from the genetic data (Fig. 3). The CE and SW groups were similar to those obtained by Flander et al. [34], which were separated by the Qinling Mountains (Fig. 1). The genetic isolation and acoustic divergence between CE and SW groups suggested that Qinling Mountains may be geographic barriers that drive acoustic divergence in RF. Flander et al. [34] only collected one individual in NE China, while in our study we collected multiple samples from three populations in northeast China. The NE populations formed a monophyletic lineage with the CE populations. Therefore, according to the divergence of lineages of *R. ferrumequinum*, we studied the acoustic geographic variation in the total range and CE/NE region. Using this, we attempted to identify the drivers of frequency divergence of *R. ferrumequinum* across different spatial scales.

### The genetic drift hypothesis

A correlation between acoustic and genetic distances indicates that the observed pattern of geographic variation could be largely due to genetic drift. Several studies have demonstrated that genetic drift can lead to acoustic divergences in some animals [5], [17]. However, in our study, differences in RF were not likely due to genetic drift. Our results showed that RF variation was not correlated with genetic divergence, either in the entire range we studied or in the CE/NE region (Table 5). The genetic distance between CE and NE (mean: 0.068±0.0058) suggested they were closely related, but the RF divergence for these two groups was large (ca. 7 kHz). In contrast, the SW and CE comparison showed a larger genetic distance (mean: 0.087±0.0062), but a relatively low RF divergence (ca. 3 kHz). Thus, it is not likely that the geographic variation in RF has been affected by genetic drift.

### The cultural drift hypothesis

Cultural transmission and copying errors are major drivers of stochastic divergence in learned vocal signals [67], [68]. In other bats, cultural drift has been suggested to yield variation in call frequency [2], [6], [22]. In this study, since geographic variation in RF showed a significantly positive correlation with geographic and longitudinal variation in the entire range we examined and the CE/NE region (Table 5), this supports the hypothesis that the observed pattern of geographic variation is likely due, at least in part, to cultural drift.

One factor that may drive cultural drift is mother-offspring transmission [6]. In Yoshino et al. [6] the maternal transmission hypothesis was supported due to a much higher correlation with maternally inherited genetic markers than nuclear markers. In *R. ferrumequinum*, the RF of a developing infant is influenced in part by that of its mother [27], suggesting maternal transmission could be important. However, the RF variation of *R. ferrumequinum* had a low correlation with both mtDNA and nDNA, and that of mtDNA was even lower than that of nDNA (Table 5). These results suggested that, while cultural drift may be occurring, mother-offspring transmission of RF was not the dominant driver of cultural drift. Instead horizontal learning [69], [70] among group members might shape learned signal convergence in *R. ferrumequinum*, which would imply that the social effect (facilitating group cohesion or the exclusion of nongroup members) of the group vocal signature might be greater than the effect of the vocal transmission of from mother to offspring.

### The acoustic adaptation hypothesis

In addition to cultural drift, environmental selection pressure might also contribute to RF variation. Both temperature and humidity have a direct influence on sound transmission and absorption [5], [71] and each alter the effect of the other on ultrasound attenuation rates under certain conditions [31]. Thus, these climatic factors are likely to be important in driving acoustic divergence in *R. ferrumequinum.* While this does not account for other environmental factors that may impact sound propagation, such as vegetation types, ambient noise and air turbulence [72], [73], [74], it provides an initial test of whether ecological selection might be important.

In our study, we found a positive correlation between acoustic and MAT distance, controlling for latitudinal or longitudinal distance, which is consistent with those found in a terrestrial muroid rodent [5]. The CE has a monsoon climate that results in higher average temperatures than is found in the NE and SW, which may explain the higher RF in this region (Fig. 2A). However, we did not detect the effect of MAPW and MARH on RF of echolocation calls within *R. ferrumequinum* region (Table 5). Studies in other bat species have shown varying roles of precipitation and humidity, with some studies agreeing with ours in finding no relationship [31], while others have found a positive or negative relationship [12], [13], [29]. Overall, our data suggested that ecological selection (to temperature, but not moisture) contributed, or is correlated with factors that contributed to the observed geographic variation in the RF of *R. ferrumequinum*, though we recognize there may be other ecological variables, such as habitat structure, that are important as well.

## Conclusions

Our study demonstrates significant geographic differences in RF of *R. ferrumequinum* in China, which corresponded to three phylogenetic groups identified from genetic data. By integrating genetic structure, geographic variation, and environmental differences, it appeared that divergence in *R. ferrumequinum* calls across China were most likely due to cultural drift and ecological selection, but not genetic drift. These results were consistent with other studies that have examined geographic variation in calls (e.g., [2], [12], [13]) in suggesting there is typically more than one selective force driving divergences among calls.
